# Molecular Imaging of Tumors Using a Quantitative *T*_1_ Mapping Technique via Magnetic Resonance Imaging

**DOI:** 10.3390/diagnostics5030318

**Published:** 2015-07-17

**Authors:** Kelsey Herrmann, Mette L. Johansen, Sonya E. Craig, Jason Vincent, Michael Howell, Ying Gao, Lan Lu, Bernadette Erokwu, Richard S. Agnes, Zheng-Rong Lu, Jonathan K. Pokorski, James Basilion, Vikas Gulani, Mark Griswold, Chris Flask, Susann M. Brady-Kalnay

**Affiliations:** 1 Department of Neurosciences, School of Medicine, Case Western Reserve University, Cleveland, OH 44106, USA; E-Mail: kelsey.herrmann@case.edu; 2 Department of Molecular Biology and Microbiology, School of Medicine, Case Western Reserve University, Cleveland, OH 44106, USA; E-Mails: mette.johansen@case.edu (M.L.J.); sonya.ensslen@case.edu (S.E.C.); jason.vincent@case.edu (J.V.); michael.howell@case.edu (M.H.); 3 Department of Biomedical Engineering, Case Western Reserve University, Cleveland, OH 44106, USA; E-Mails: ying.gao@case.edu (Y.G.); zheng-rong.lu@case.edu (Z.-R.L.); james.basilion@case.edu (J.B.); vikas.gulani@case.edu (V.G.); mark.griswold@case.edu (M.G.); christopher.flask@case.edu (C.F.); 4 Department of Radiology, School of Medicine, Case Western Reserve University, Cleveland, OH 44106, USA; E-Mails: lan.lu@case.edu (L.L.); bernadette.erokwu@case.edu (B.E.); 5 Department of Urology, Case Western Reserve University, Cleveland, OH 44106, USA; 6 Department of Macromolecular Science and Engineering, Case Western Reserve University, Cleveland, OH 44106, USA; E-Mails: richard.agnes@case.edu (R.S.A.); jon.pokorski@case.edu (J.K.P.); 7 The National Foundation for Cancer Research (NFCR) Center for Molecular Imaging, Case Western Reserve University, Cleveland, OH 44106, USA; 8 Department of Pediatrics, Case Western Reserve University, Cleveland, OH 44106, USA

**Keywords:** magnetic resonance imaging, molecular imaging, *T*_1_ relaxation time, cancer imaging, tumor detection, protein tyrosine phosphatase, PTPmu

## Abstract

Magnetic resonance imaging (MRI) of glioblastoma multiforme (GBM) with molecular imaging agents would allow for the specific localization of brain tumors. Prior studies using *T*_1_-weighted MR imaging demonstrated that the SBK2-Tris-(Gd-DOTA)_3_ molecular imaging agent labeled heterotopic xenograft models of brain tumors more intensely than non-specific contrast agents using conventional *T*_1_-weighted imaging techniques. In this study, we used a dynamic quantitative *T*_1_ mapping strategy to more objectively compare intra-tumoral retention of the SBK2-Tris-(Gd-DOTA)_3_ agent over time in comparison to non-targeted control agents. Our results demonstrate that the targeted SBK2-Tris-(Gd-DOTA)_3_ agent, a scrambled-Tris-(Gd-DOTA)_3_ control agent, and the non-specific clinical contrast agent Optimark™ all enhanced flank tumors of human glioma cells with similar maximal changes on *T*_1_ mapping. However, the retention of the agents differs. The non-specific agents show significant recovery within 20 min by an increase in *T*_1_ while the specific agent SBK2-Tris-(Gd-DOTA)_3_ is retained in the tumors and shows little recovery over 60 min. The retention effect is demonstrated by percent change in *T*_1_ values and slope calculations as well as by calculations of gadolinium concentration in tumor compared to muscle. Quantitative *T*_1_ mapping demonstrates the superior binding and retention in tumors of the SBK2-Tris-(Gd-DOTA)_3_ agent over time compared to the non-specific contrast agent currently in clinical use.

## 1. Introduction

While various imaging modalities are used in clinical and surgical settings, magnetic resonance imaging (MRI) is the preferred method of brain tumor imaging prior to surgery. The benefits of MRI include excellent delineation of anatomic detail and multiple available soft tissue contrast mechanisms. To improve MRI assessments of tumors, targeted molecular contrast agents are in development to provide biological specificity in identifying and distinguishing the irregular and indistinct tumor margins of invasive cancers [1,2]. A typical goal for molecular contrast agents is to bind preferentially to molecules specific to the tumor in comparison to the surrounding normal tissue. Therefore, molecules that are enriched in the tumor microenvironment are ideal targets.

Glioblastoma multiforme (GBM) is a highly aggressive tumor that arises in the brain. Patients with GBM survive, on average, one year post-diagnosis [3] despite undergoing surgery, radiation, and chemotherapy. The devastating nature of GBM is due to the highly dispersive and invasive tumor cells that infiltrate the brain and migrate away from the main tumor mass. These distant, migratory cells, often undetectable by conventional MRI methods, make complete surgical resection difficult [4]. The receptor protein tyrosine phosphatase PTPµ is a transmembrane protein that is proteolyzed in tumor tissue to yield an extracellular fragment and a membrane-freed intracellular fragment [5,6]. The proteolyzed extracellular fragment of PTPµ accumulates in aggressive GBM tumors and provides a detectable moiety for molecular imaging [7]. 

We have previously developed a molecular imaging agent that specifically binds to the PTPµ extracellular fragment, called SBK2 that is linked to a fluorophore, and labels both the main GBM tumor mass [5] and greater than 99% of the dispersing cells up to 3.5 mm away from the main tumor [7]. When conjugated to a gadolinium (Gd) chelate as an MRI contrast agent, the SBK2-Tris-(Gd-DOTA)_3_ agent showed greater contrast enhancement than the non-specific agent, ProHance, when intravenously injected into mice bearing heterotopic flank tumors of human glioma cells using conventional *T*_1_-weighted MR imaging [8]. The SBK2-Tris-(Gd-DOTA)_3_ agent labeled the tumors within 5 min with a high level of contrast persisting for 2 h post injection, significantly higher than ProHance^®^ alone [8]. One limitation of these prior studies is that conventional *T*_1_-weighted imaging techniques rely on relative signal intensity changes over time and are inherently qualitative at each time point. The *T*_1_-weighted values of a given region of interest are relative to the values from another area. For example, a comparison of tumor to adjacent muscle at that particular time point is represented as a contrast to noise value for a particular time point. Therefore despite these promising initial results, a rigorously quantitative approach was needed to determine the *in vivo* binding and retention properties of the SBK2-Tris-(Gd-DOTA)_3_ agent in comparison to controls over time.

In contrast to *T*_1_-weighted imaging, *T*_1_ relaxation time mapping is a quantitative approach that allows measurement of *T*_1_ values. When acquired dynamically, these *T*_1_ relaxation time values allow each time point to be objectively, quantitatively, and longitudinally compared within a single agent as well as among different agents. Furthermore, *T*_1_ mapping limits the impact of scanner dependent variation. Quantitative *T*_1_ measurements can then be used to calculate contrast agent concentration within each imaging voxel (volume element). This technique is especially important for molecular imaging where the goal is to relate regions of accumulating and/or retained contrast agent to the location of specific disease markers. We hypothesized that *T*_1_ mapping could be performed to quantitatively determine contrast agent concentration in tumors as well as evaluate successful specific targeting by contrast agents. Therefore, we conducted a series of experiments comparing the SBK2-Tris-(Gd-DOTA)_3_ agent to the scrambled-Tris-(Gd-DOTA)_3_ control as well as a non-specific clinical contrast agent, Optimark™, in mice bearing heterotopic flank tumors of human LN-229 glioma cells. In this study, we dynamically evaluated the extent and duration of tumor enhancement following contrast administration with *T*_1_ mapping. We observed that all of the contrast agents achieve similar levels of initial *T*_1_ reductions indicating similar delivery to the tumor region between 10 to 15 min after injection. Notably, a large and statistically significant difference was observed in the retention of the specific SBK2-Tris-(Gd-DOTA)_3_ agent *versus* the non-specific agents at later time points. We conclude that when evaluating and comparing MR molecular imaging agents, evaluation of retention time as determined with *T*_1_ mapping is a robust indicator of agent specificity.

## 2. Methods and Materials

All reagents were used without further purification unless otherwise stated. Optimark™ was purchased from Mallinckrodt Pharmaceuticals (St. Louis, MO, USA), and saline was obtained from Hospira, Inc. (Lake Forest, IL, USA). The Fmoc-protected amino acids, 2-chlorotrityl chloride resin, and benzotriazol-1-yl-oxy-tris-(pyrrolidino) phosphonium hexafluorophosphate (PyBOP) used for peptide synthesis were purchased from Chem-Impex International, Inc. (Wood Dale, IL, USA), along with anhydrous *N*,*N*-diisopropylethyl amine (DIPEA), trifluoroacetic acid (TFA), 1,2-diethanethiol, triisopropylsilane and piperidine from Sigma-Aldrich (St. Louis, MO, USA). *N*,*N-*dimethylformamide (DMF), and dichloromethane were purchased from Fisher Scientific (Pittsburgh, PA, USA). Anhydrous 1-hydroxybenzotriazole (HOBt) was obtained from Apex Bio Technology (Houston, TX, USA). The detailed syntheses of maleimido-tris-propargyl [9] and azido-(Gd-DOTA) [10] have been described previously. Gadolinium (III) acetate tetrahydrate was from Strem Chemicals (Newburyport, MA, USA).

### 2.1. Synthesis and Characterization of SBK2-Tris-(Gd-DOTA)_3_ and Scrambled-Tris-(Gd-DOTA)_3_ Agents

The syntheses and characterization of SBK2-Tris-(Gd-DOTA)_3_ and scrambled-Tris-(Gd-DOTA)_3_ agents have been described previously [8]. Briefly, the peptides were synthesized using conventional solid-phase synthetic methods and FMOC-protected amino acids. Peptide purity was assessed using LC-MS/MS on a Thermo Finnigan LTQ Linear ion trap mass spectrometer with a Phenomenex Jupiter C18 reversed-phase capillary chromatography column. Peptides were conjugated to maleimido-tris-propargyl by reacting the N-terminal cysteine with the maleimide group. A copper-catalyzed azide-alkyne cycloaddition reaction was then used to couple azido-(Gd-DOTA) to the free alkyne groups of the tris-propargyl peptides. Matrix-assisted laser desorption/ionization time-of-flight (MALDI-TOF; Autoflex Speed, Bruker Corp., Billerica, MA, USA) was used to monitor the conjugation reaction of azido-(Gd-DOTA) to the peptide-tris-propargyl moieties. Gadolinium (Gd) content was measured using inductively coupled plasma optical emission spectroscopy (ICP-OES) (Agilent 730 Axial ICP-OES; Agilent Technologies, Wilmington, DE, USA). *T*_1_ relaxation constants for the agents were measured on the Bruker Biospec 9.4T MRI scanner (Bruker Corp., Billerica, MA, USA) at 37 °C.

### 2.2. Cell Culture and Flank Tumor Implants

The human LN-229 glioma cell line was purchased from American Type Culture Collection (Manassas, VA, USA) and cultured in DMEM medium supplemented with 5% fetal bovine serum. Cells were infected with lentivirus encoding green fluorescent protein (GFP) and stable cultures were established. The cells were diluted in a 1:1 mixture of PBS and BD Matrigel™ Matrix (BD Biosciences, Franklin Lakes, NJ, USA), and injected into the right flank of both male and female nude athymic mice (NCr-nu/+, NCr-nu/nu, 20–25 g each) as previously described [5]. Each flank was implanted with 2 × 10^6^ cells. To correlate tumor position with GFP fluorescence, mice were imaged using the Perkin-Elmer Maestro™ FLEX *In Vivo* Imaging System as previously described [5]. 

### 2.3. Molecular Imaging of Tumors with MRI

The MRI study was performed using a Bruker Biospec 9.4 T preclinical MRI scanner (Bruker Corp., Billerica, MA, USA) with a 35 mm inner diameter mouse body radio frequency (RF) coil. Mice bearing LN-229 flank tumors were imaged at 2–5 weeks post tumor implant. Polyurethane tubing (0.014″ ID × 0.033″ OD) (SAI Infusion Technologies, Lake Villa, IL, USA) was connected to a 1 mL syringe and loaded with the appropriate amount of a given contrast agent dissolved or diluted in saline. All agents were administered an equal Gd concentration of 0.2 mmol·Gd/kg. The total volume of each agent injected was <100 μL, with an additional 50 μL of saline used to flush the line and ensure that the full dose was administered to the animal. After mice were anesthetized with a 2% isoflurane-oxygen mixture in an isoflurane induction chamber, tail veins were catheterized with a 26 gauge veterinary catheter and connected to the pre-loaded tubing described above. The animals were moved into the magnet and kept under inhalation anesthesia with 1.5% isoflurane-oxygen via a nose cone. A respiratory sensor connected to a monitoring system (SA Instruments, Stony Brook, NY, USA) was placed on the back of the animal to monitor rate and depth of respiration. Body temperature was maintained at 35 ± 1 °C by blowing warm air into the magnet through a feedback control system. A group of 5–6 mice was used for each agent. 

High-resolution *T*_2_-weighted images were first obtained for each mouse using a RARE (Rapid Acquisition with Relaxation Enhancement) acquisition (TR/TE = 5000/40 ms, 20 slices, resolution = 0.117 × 0.117 × 0.5 mm) to select the imaging slice for the dynamic *T*_1_ mapping acquisition [11]. The dynamic *T*_1_ data were then acquired using a snapshot GRE (Gradient Recalled Echo) acquisition with inversion recovery preparation described previously [12,13] (10 inversion times (263, 775, 1287, 1799, 2311, 2823, 3335, 3847, 4359, and 4871 ms), GRE imaging readout TR/TE = 4.0 ms/1.3 ms, flip angle = 10 degrees, resolution = 0.234 × 0.234 × 1 mm, Field of View (FOV) = 30 × 30 mm, and 10 signal averages). The total acquisition for each *T*_1_ mapping scan was 2.5 min. After five baseline/pre-injection *T*_1_ mapping scans, Optimark™, the targeted SBK2-Tris-(Gd-DOTA)_3_ agent, or the non-targeted scrambled-Tris-(Gd-DOTA)_3_ control was injected at a dose of 0.2 mmol of Gd/kg followed by a 50 µL flush of saline. *T*_1_ maps were then consecutively acquired every 2.5 min over 62.5 min.

### 2.4. Calculation of T_1_ Mapping Values, Gd Concentration, and Slope Analysis

The MRI data were imported into MATLAB enabling estimation of both pixel-wise *T*_1_ relaxation time maps as well as mean intra-tumoral *T*_1_ changes using an ROI analysis. The *T*_1_ maps were obtained from the *T*_1_ mapping acquisition using previously described methods based on mono-exponential models [13]. To compare the multiple imaging agents, the pre- and post-contrast *T*_1_ maps were then used to calculate maps of % change in *T*_1_, which is directly related to the concentration of each agent within the tumor. To calculate Gd concentrations, *T*_1_ relaxivity constants determined at 9.4T were used along with *T*_1_ map values.

As the *T*_1_ maps and *T*_2_-weighted images were co-registered, the ROI analysis was performed by manually drawing the ROI on the *T*_2_-weighted images. The same ROI was then applied to all of the *T*_1_ mapping images and the average value in the ROI was calculated as a measure of tumor uptake of each imaging agent. Plots of normalized *T*_1_ values in the tumor were calculated by dividing the post-contrast injection *T*_1_ maps by an average of the 5 baseline (pre-contrast injection) tumor *T*_1_ values. This normalization was performed to limit the effects of variation in the pre-contrast tumor *T*_1_ values on the comparison of the molecular imaging agents.

As an imaging marker for contrast agent retention, a slope analysis was performed on the normalized *T*_1_ values. Slopes were calculated for Optimark™, scrambled-Tris-(Gd-DOTA)_3_, and SBK2-Tris-(Gd-DOTA)_3_ by using all of the mean tumor *T*_1_ values from 15 to 60 min following agent administration. For each pair of contrast agents, normalized *T*_1_ map values at each time point, along with the slopes of post-contrast *T*_1_ curves were evaluated for statistical significance using a two-tailed Student’s *t*-test, assuming statistical significance at *p* < 0.05. An *F*-test was also used to determine if the variance was significantly different between the two samples being compared.

## 3. Results

### 3.1. Quantitative MRI Contrast Agent Evaluation

**Figure 1:**
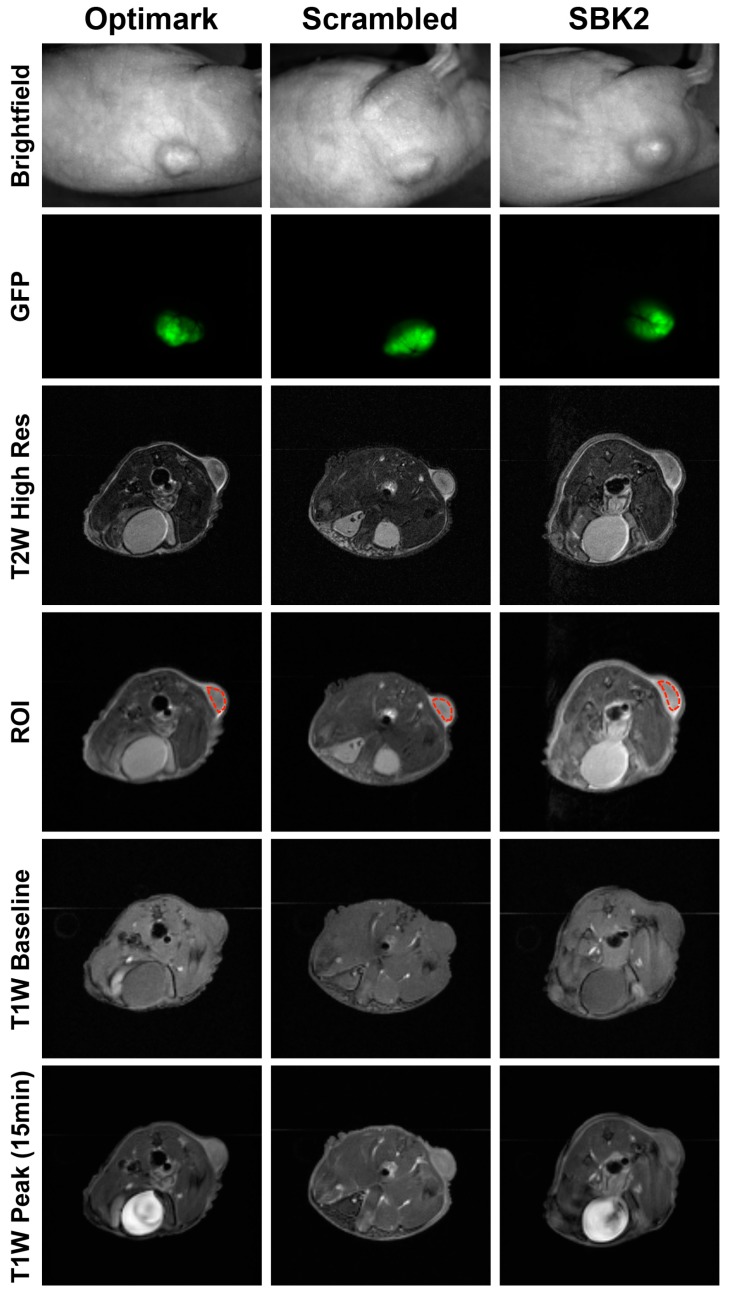
The SBK2-Tris-(Gd-DOTA)_3_ molecular imaging agent and the non-specific agents all enhance LN-229 tumors. Representative brightfield image of GFP-positive LN-229 flank tumors for animals where 0.2 mmol Gd/kg of Optimark™, scrambled-Tris-(Gd-DOTA)_3_, or SBK2-Tris-(Gd-DOTA)_3_ was administered. *N* = 6 for Optimark™, *N* = 5 for scrambled-Tris-(Gd-DOTA)_3_, and *N* = 5 for SBK2-Tris-(Gd-DOTA)_3_. GFP fluorescence image of LN-229 tumor cells for each of the three contrast agents. *T*_2_ low-resolution images with Region of Interest (ROI), illustrated by a dashed red line, show the tumor area used for *T*_1_ map quantification in Figure 2, Figure 3 and Figure 4. Axial *T*_1_-weighted images of LN-229 flank tumor at baseline (before injection of contrast agents) and at time of maximum contrast (15 min) following intravenous injection.

To evaluate the efficacy of the specific agent, SBK2-Tris-(Gd-DOTA)_3_, we compared this targeted agent to the scrambled-Tris-(Gd-DOTA)_3_, and the non-specific conventional clinical contrast agent Optimark™. We acquired and analyzed 2D axial *T*_1_ mapping images in GFP-positive LN-229 human glioma flank tumors heterotopically implanted in athymic (nu/nu) mice at 2–5 weeks post-implantation. Five *T*_1_ maps of the tumor region were acquired before injection of contrast agents to serve as baseline measurements, and then at 2.5 min intervals for 62.5 min following intravenous injection of Optimark™, scrambled-Tris-(Gd-DOTA)_3_, or SBK2-Tris-(Gd-DOTA)_3_ agents administered at a dose of 0.2 mmol·Gd/kg. Representative fluorescence and MRI images of mice with the implanted flank tumors are shown in Figure 1 for each contrast agent. Using optical imaging, the physical outline of the flank tumors is evident under brightfield illumination (Figure 1) and the GFP-positive LN-229 glioma cells are also clearly visible in the fluorescence images (Figure 1). For MR imaging, the axial *T*_2_-weighted images (Figure 1) are shown with the regions of interest (ROIs) indicated by dashed lines that were used for *T*_1_ mapping. In this view, the flank tumors are clearly visible in the *T*_2_-weighted images allowing for accurate ROI selection. Figure 1 also shows *T*_1_-weighted images acquired from the *T*_1_ mapping acquisitions both at baseline (Figure 1) and 15 min following contrast agent injection (Figure 1), respectively. All three contrast agents showed significant contrast uptake as evidenced by the relative change in the *T*_1_-weighted images. However, a rigorous quantitative comparison is not possible with these *T*_1_-weighted images.

### 3.2. Quantitative T_1_ Mapping of MRI Contrast Agent Efficacy in Heterotopic Glioma Flank Tumors

**Figure 2:**
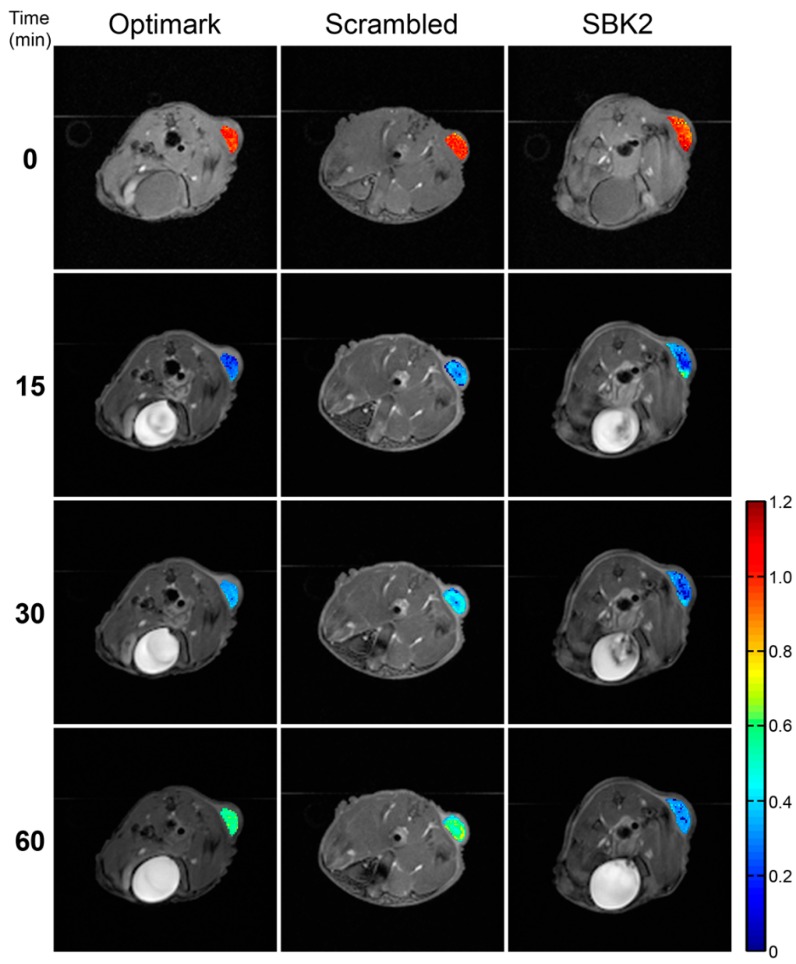
The specific molecular imaging agent SBK2-Tris-(Gd-DOTA)_3_ results in prolonged decrease in *T*_1_ relaxation time in tumors compared to non-specific agents. Normalized *T*_1_ maps of the flank tumors overlaid onto *T*_1_-weighted images at pre-contrast (0 min); 15 min post-injection; 30 min post-injection; and 60 min post-injection. The color-coded scale bar indicates normalized *T*_1_ relaxation time values with dark blue representing the lowest *T*_1_ values resulting from the *T*_1_ shortening effect of the contrast agents. Note the prolonged decrease in normalized *T*_1_ values with the SBK2-Tris-(Gd-DOTA)_3_ agent resulting in lower *T*_1_ map values while the *T*_1_ values of the non-specific agents have returned to about 60% of baseline.

To obtain a quantitative comparison of the difference in tumor enhancement over time between the three contrast agents, *T*_1_ mapping was employed to compare the changes in *T*_1_ relaxation times in the tumor for each contrast agent over time. Pixel-wise maps of *T*_1_ relaxation time were normalized to the mean baseline *T*_1_ values and are shown as heat maps overlaying the corresponding gray scale axial images for the indicated time points in Figure 2. The color-coded scale bar indicates the normalized *T*_1_ relaxation time from lowest (blue) to highest (red). As expected, all of the contrast agents resulted in a reduction in *T*_1_ relaxation time within the first 15 min after agent injection. Importantly, by 30 min post-injection, both the Optimark™ and scrambled-Tris-(Gd-DOTA)_3_ agents had started to clear from the tumor while the targeted SBK2-Tris-(Gd-DOTA)_3_ contrast agent was retained within the tumor as evidenced by the limited change in *T*_1_ relaxation time relative to that observed at 15 min post-injection (Figure 2). At one-hour post-injection, the normalized *T*_1_ relaxation time had returned to ~60% of the pre-contrast values for both Optimark™ and scrambled-Tris-(Gd-DOTA)_3_ agents (Figure 2).

**Figure 3:**
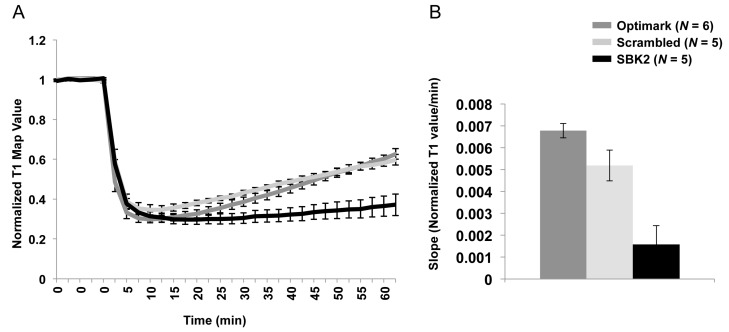
Mean tumor normalized *T*_1_ values and slope analysis following intravenous administration of Optimark™, scrambled-Tris-(Gd-DOTA)_3_, or SBK2-Tris-(Gd-DOTA)_3_ contrast agents in cohorts of nu/nu athymic mice bearing glioma flank tumors administered at a dose of 0.2 mmol·Gd/kg. Note the sustained decrease in normalized *T*_1_ for SBK2-Tris-(Gd-DOTA)_3_ as well as the significant difference in slope due to agent clearance between the non-specific agents compared to SBK2-Tris-(Gd-DOTA)_3_, which showed the highest retention. *N* = 6 for Optimark™, *N* = 5 for scrambled-Tris-(Gd-DOTA)_3_, and *N* = 5 for SBK2-Tris-(Gd-DOTA)_3_. Data plotted as means ± standard error. (**A**) Mean tumor normalized *T*_1_ values at baseline and after agent injection measured every 2.5 min for 62.5 min. Normalized *T*_1_ values are significantly different between SBK2-Tris-(Gd-DOTA)_3_ and Optimark™ from 30–62.5 min (ranges from *p* < 0.001 to *p* < 0.04 depending upon the time point), and between SBK2-Tris-(Gd-DOTA)_3_ and scrambled-Tris-(Gd-DOTA)_3_ from 17.5–62.5 min (ranges from *p* < 0.002 to *p* < 0.03). Optimark™ and scrambled-Tris-(Gd-DOTA)_3_ were not significantly different at any time point. (**B**) The slopes of the lines were determined between 15 and 60 min post-injection to examine the rate of agent clearance. The slope of SBK2-Tris-(Gd-DOTA)_3_ recovery was significantly different than that of both Optimark™ (*p* < 0.0002) and scrambled-Tris-(Gd-DOTA)_3_ (*p* < 0.01). The slopes of Optimark™ and scrambled-Tris-(Gd-DOTA)_3_ were not significantly different from one another.

To evaluate the retention of the agents in greater detail, we plotted the mean tumor normalized *T*_1_ values for groups of mice administered each contrast agent over the entire scanning session of 62.5 min. We found that the mean tumor normalized *T*_1_ values of all three agents showed maximal decreases in *T*_1_ between 10 and 15 min following injection (Figure 3). Importantly, the normalized *T*_1_ values for the SBK2-Tris-(Gd-DOTA)_3_ agent remain reduced over the entire 62.5 min scanning session (Figure 3), while the scrambled-Tris-(Gd-DOTA)_3_ and Optimark™ normalized *T*_1_ mapping values begin to return towards baseline levels starting at approximately 20 min. Normalized *T*_1_ values are significantly different between SBK2-Tris-(Gd-DOTA)_3_ and Optimark™ from 30–62.5 min (ranges from *p* < 0.001 to *p* < 0.04 depending upon the time point), and between SBK2-Tris-(Gd-DOTA)_3_ and scrambled-Tris-(Gd-DOTA)_3_ from 17.5–62.5 min (ranges from *p* < 0.002 to *p* < 0.03). The normalized *T*_1_ values for the Optimark™ and scrambled-Tris-(Gd-DOTA)_3_ agents were not significantly different at any time point. The rate of contrast agent clearance from the tumor was calculated by determining the change in mean tumor normalized *T*_1_ over time from 15 to 60 min for each agent. The recovery slopes of scrambled-Tris-(Gd-DOTA)_3_ and Optimark™ were not statistically different from one another. In contrast, the slope of SBK2-Tris-(Gd-DOTA)_3_ recovery differed significantly from that of both scrambled-Tris-(Gd-DOTA)_3_ (*p* < 0.01) and Optimark™ (*p* < 0.0002) demonstrating quantitatively that SBK2-Tris-(Gd-DOTA)_3_ is specifically retained in the flank tumors over time.

**Figure 4:**
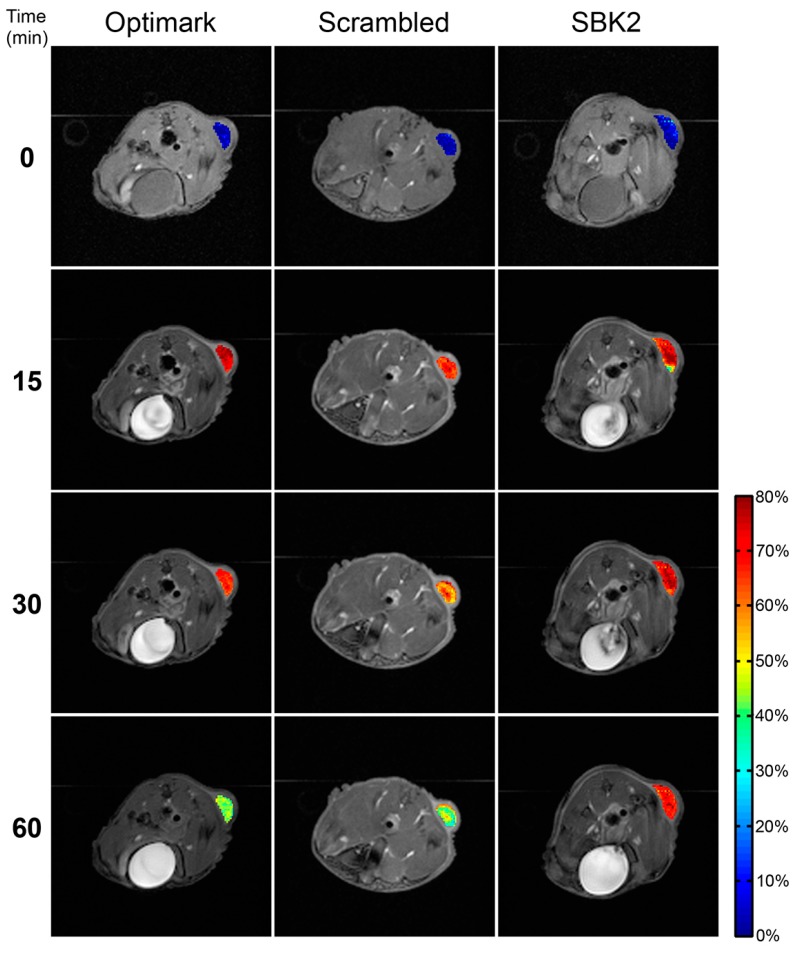
Maps of percent change in *T*_1_ relaxation time for flank tumors overlaid onto axial *T*_1_-weighted images plotted at pre-contrast (0 min); 15 min post-injection; 30 min post-injection; and 60 min post-injection. The percent change in *T*_1_ values demonstrate that the agent SBK2-Tris-(Gd-DOTA)_3_ is retained in the tumor for a longer period of time than either Optimark™ or scrambled-Tris-(Gd-DOTA)_3_ agents. The color scale indicates percent change in *T*_1_ relaxation time on a 0%–80% scale.

To complement the data presented in Figure 2 and Figure 3, maps of the percent change in *T*_1_ values were also calculated at multiple time points for the flank tumors (Figure 4). Baseline (pre-contrast) values are shown in Figure 4 and are near zero, as expected. As anticipated, the percent change in *T*_1_ at 15 min post-injection is dramatically increased for all three agents (Figure 4).

By 30 min, the percent change in *T*_1_ is showing some initial reduction for the Optimark™ and scrambled-Tris-(Gd-DOTA)_3_ agents, while the percent change in *T*_1_ for the SBK2-Tris-(Gd-DOTA)_3_ remains essentially unchanged from that at 15 min (Figure 4). By 60 min post-injection, the percent change in *T*_1_ values for Optimark™ and scrambled-Tris-(Gd-DOTA)_3_-treated animals is closer to that observed at baseline. In contrast, percent change in *T*_1_ values in the animal administered SBK2-Tris-(Gd-DOTA)_3_ remains virtually unchanged in comparison to the 15 and 30 min time points (Figure 4).

**Figure 5:**
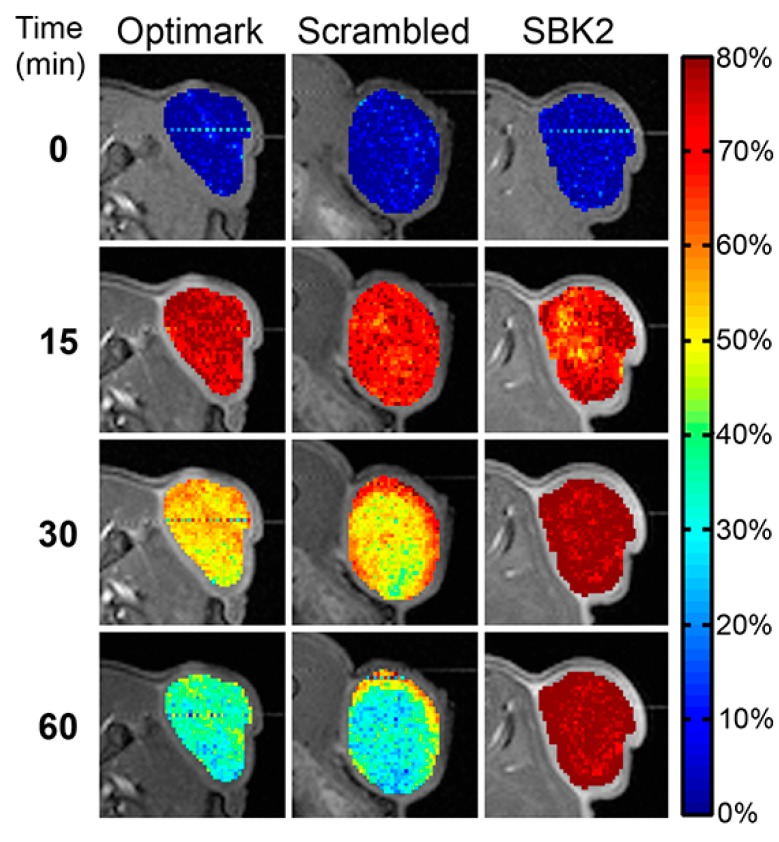
Maps representing the percent change in *T*_1_ values indicates that the tumors of mice at pre-contrast (0 min); 15 min post-injection; 30 min post-injection; and 60 min post-injection show SBK2-Tris-(Gd-DOTA)_3_ is retained for a much longer period of time than Optimark™ and scrambled-Tris-(Gd-DOTA)_3_ agents even in larger tumors. Note also that the *T*_1_ changes at 30 min and 60 min for the SBK2-Tris-(Gd-DOTA)_3_ agent are uniformly distributed throughout the tumor while the non-specific agents show rim enhancement typical of conventional agents. The color scale indicates percent change in *T*_1_ relaxation time on a 0%–80% scale.

A second group of heterotopic glioma flank tumor bearing mice with larger tumors were also administered Optimark™, scrambled-Tris-(Gd-DOTA)_3_, and SBK2-Tris-(Gd-DOTA)_3_ agents as shown in Figure 5 with enlarged images. These images show the non-heterogeneous labeling of the tumors by the non-specific agents, whereas the SBK2-Tris-(Gd-DOTA)_3_ agent uniformly recognizes the entire tumor. The “rim” or “edge” effect of the non-specific agents is often seen in clinical imaging [14]. A similar result demonstrating this “rim” effect for non-specific agents was also observed in our previous studies [8]. In contrast, the SBK2-Tris-(Gd-DOTA)_3_ agent exhibits remarkable uniformity across the entire tumor consistent with binding of the PTPµ fragment within the tumors rather than in just the tumor (neo)vasculature.

**Figure 6:**
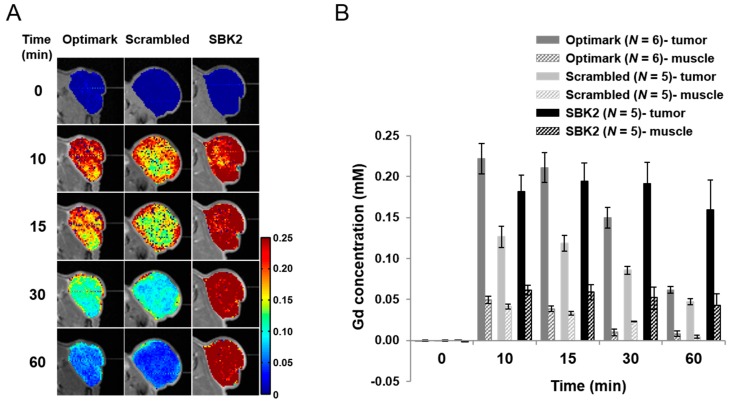
Gadolinium concentrations in tumor and muscle of animals treated with different contrast agents. (**A**) Maps of gadolinium concentration overlaid onto axial *T*_1_-weighted images plotted at pre-contrast (0 min); 10 min post-injection; 15 min post-injection; 30 min post-injection; and 60 min post-injection. Consistent with *T*_1_ map values observed for the non-specific contrast agents, Gd concentrations are highest at 10 and 15 min in tumors of animals treated with Optimark™ and scrambled-Tris-(Gd-DOTA)_3_, and then rapidly decrease at later time points. Gd concentration in tumor of animal receiving SBK2-Tris-(Gd-DOTA)_3_ remains at near peak levels at 60 min indicating retention of the agent in the tumor. (**B**) Mean gadolinium concentrations ± SE are plotted for tumor and muscle for groups of animals treated with the indicated contrast agents at different time points. Gd concentrations in tumors of animals treated with Optimark™ and scrambled-Tris-(Gd-DOTA)_3_ are highest at 10 and 15 min after injection and then decline. In contrast, the Gd concentration in tumors of animals treated with SBK2-Tris-(Gd-DOTA)_3_ persist at approximately 0.15 mM from 10 to 60 min. Gd concentrations calculated in control muscle regions (hatched bars) are substantially lower than those in tumors.

A significant benefit of *T*_1_ mapping analysis is that this quantitative imaging method allows for the calculation of contrast agent concentration in any region of interest. As shown in Figure 6, the Gd content in tumors of animals treated with the specific SBK2-Tris-(Gd-DOTA)_3_ agent remains at peak levels even an hour after treatment while clearance begins to occur before 30 min in the animals treated with the non-specific agents. As expected, the Gd concentration in control areas of muscle is much lower than that of tumor (Figure 6B).

## 4. Discussion and Conclusion

We dynamically evaluated the extent and duration of tumor enhancement following contrast administration with *T*_1_ mapping. Our study using *T*_1_ mapping to evaluate the efficacy of the SBK2-Tris-(Gd-DOTA)_3_ molecular imaging agent in flank GBM tumors demonstrates that SBK2-Tris-(Gd-DOTA)_3_ is effective at specifically recognizing and binding GBM tumors compared to non-targeted contrast agents. Three major findings come to light as a result of our study.

The first major finding is that the *T*_1_ mapping method can be used to dynamically measure *T*_1_ values and determine Gd-based contrast agent concentration over time in tumors using molecular imaging agents. We observed that all of the contrast agents achieve similar levels of initial reductions in *T*_1_ relaxation times indicating comparable delivery to the tumor region between 10 to 15 min after injection. Notably, a large and statistically significant difference was observed in the duration of the reduction in *T*_1_ relaxation time and the retention of the specific SBK2-Tris-(Gd-DOTA)_3_ agent *versus* the non-specific agents. Whereas the *T*_1_ relaxation times for the non-specific contrast agents more rapidly returned towards their respective pre-contrast levels, the *T*_1_ relaxation times for the SBK2-Tris-(Gd-DOTA)_3_ agent remained close to the maximum change in *T*_1_ relaxation for the entire hour of imaging (Figure 2 and Figure 3). Importantly, *T*_1_ mapping provides improved quantification in comparison to conventional *T*_1_-weighted imaging. In *T*_1_-weighted imaging, the accumulation of contrast agent is associated with increased signal intensity in the *T*_1_-weighted images due to the *T*_1_ relaxation time reductions imposed by the contrast agent. Unfortunately, the signal intensity changes detected in *T*_1_-weighted imaging studies are not quantitative in nature as these signals are also impacted by many other factors including variation in the excitation and detection efficiency of the MRI coils of different scanners. Therefore, quantitative *T*_1_-weighted imaging studies require modeling of the signal intensity profile. As a result, the individual time points in a *T*_1_-weighted study are only qualitative by nature. As shown herein, dynamic *T*_1_ mapping provides quantitative assessments at each imaging time point providing the opportunity to assess both the delivery and retention of the contrast agents (*i.e.*, minimum *T*_1_ relaxation time; maximal change in *T*_1_) as shown in Figure 3. These data demonstrate the utility of quantitative *T*_1_ mapping as opposed to conventional *T*_1_-weighted imaging in objectively quantifying the ability of a targeted molecular agent to label tumors and allows for the calculation of clearance rates among different agents.

The second major finding is that *T*_1_ mapping allowed us to determine that the extent of initial contrast is approximately the same regardless of agent specificity. Tumor imaging research often focuses on the enhanced permeability and retention (EPR) effect that is observed in tumor tissue due to pathophysiological changes in the vasculature and host environment that promotes the leakage of large macromolecules (>40 kD to 800 kD) into tumor tissue while simultaneously slowing the clearance of macromolecules through the lymphatic system [15]. The initial delivery of these molecules is due to the enhanced permeability of the vessels in tumors, which is supported by our data. Gd-chelates are small molecules that passively leak from the circulation into the extracellular fluid [16]. Due to their small size, Gd-chelates also rapidly diffuse back into the vasculature [17]. An actively targeted small molecular weight contrast agent conjugated to Gd, on the other hand, that specifically binds and recognizes a molecular target should be retained [16,18].

Our third major finding is consistent with the idea of prolonged tumor retention being exhibited only by specifically targeted agents such as SBK2-Tris-(Gd-DOTA)_3_. Our data show that SBK2-Tris-(Gd-DOTA)_3_, but not the non-specific agents Optimark™ and scrambled-Tris-(Gd-DOTA)_3_ produced a sustained decrease in *T*_1_ map values. As shown in Figure 3, we observed similar initial contrast due to the passive diffusion of Gd-chelates into tumor tissue. This corresponds to the greatest change in normalized *T*_1_ map values at 10 min obtained for the non-specific contrast agents that then rapidly return to baseline over the course of the hour. In our tumor model, the mean normalized *T*_1_ map or numerical value for passive targeting, occurring at 10 min, is between 0.30 for Optimark™ and 0.35 for scrambled-Tris-(Gd-DOTA)_3_. For SBK2-Tris-(Gd-DOTA)_3_, on the other hand, the normalized *T*_1_ map values stabilized at approximately the same maximal decrease for as long as the tumors were imaged, over the course of an hour. Furthermore, *T*_1_ mapping allows Gd concentration to be calculated. As shown in Figure 6, Gd concentrations reached maximal levels in tumors of animals treated with non-specific contrast agents between 10 and 15 min post-injection before decreasing, while Gd concentration in the tumors of SBK2-Tris-(Gd-DOTA)_3_-treated animals persisted at near maximal concentrations for the hour of imaging. The SBK2-Tris-(Gd-DOTA)_3_ agent binds specifically to PTPµ fragments present in glioma tumors [5,8]. Therefore, in this heterotopic tumor model, the retention of the agent within the tumor results from the specific *in vivo* binding to PTPµ fragments. Since all three contrast agents lower *T*_1_ map values to a similar extent, but differ dramatically in the period of increased Gd concentration and duration of reduced *T*_1_ map values in tumor, these data suggest that measurement of agent concentration and retention within this 30–60 min time frame is a powerful tool with which to assess specific recognition of a disease state.

Importantly, the *T*_1_ mapping technique is easily translated into evaluation of molecular imaging agents using conventional clinical scanners. We hypothesize that the increased retention of SBK2-Tris-(Gd-DOTA)_3_ in tumors will allow detection of smaller tumors as the sustained reduction in *T*_1_ values will provide the opportunity to acquire *T*_1_ maps at high resolution, which requires these longer acquisition times.
